# From Identification to Characterization of the Multiple Sclerosis Susceptibility Gene *CLEC16A*

**DOI:** 10.3390/ijms14034476

**Published:** 2013-02-25

**Authors:** Tone Berge, Ingvild Sørum Leikfoss, Hanne F. Harbo

**Affiliations:** 1 Department of Neurology, Oslo University Hospital, Ullevål, Oslo 0407, Norway; E-Mails: ingvild.sorum@medisin.uio.no (I.S.L.); h.f.harbo@medisin.uio.no (H.F.H.); 2 Department of Anatomy, Institute of Basic Medical Sciences, University of Oslo, Oslo 0317, Norway; 3 Institute of Clinical Medicine, University of Oslo, Oslo 0450, Norway

**Keywords:** autoimmunity, multiple sclerosis, *CLEC16A*, *SOCS1*, *DEXI*, *CIITA*

## Abstract

Multiple sclerosis (MS) is an inflammatory, demyelinating disorder of the central nervous system that develops in genetically susceptible individuals, probably triggered by common environmental factors. Human leukocyte antigen (HLA) loci were early shown to confer the strongest genetic associations in MS. Now, more than 50 non-HLA MS susceptibility loci are identified, of which the majority are located in immune-regulatory genes. Single nucleotide polymorphisms (SNPs) in the C-type lectin-like domain family 16A (*CLEC16A*) gene were among the first non-HLA genetic variants that were confirmed to be associated with MS. Fine-mapping has indicated a primary association in MS and also other autoimmune diseases to intronic *CLEC16A* SNPs. Here, we review the identification of MS susceptibility variants in the *CLEC16A* gene region, functional studies of the CLEC16A molecule and the recent progress in understanding the implications thereof for MS development. This may serve as an example of the importance for further molecular investigation of the loci identified in genetic studies, with the aim to translate this knowledge into the clinic.

## 1. Introduction

Multiple sclerosis (MS) is an inflammatory, demyelinating disorder of the central nervous system (CNS) that causes relapsing-remitting attacks (RR-MS) or a progressive disease (primary or secondary progressive MS) leading to different degrees of physical and cognitive disability. An increasing number of immune-modulatory medications are used in RR-MS with variable effects on relapse rate and progression of MS lesions, evaluated by magnetic resonance imaging (MRI) [1]. There is a global, latitudinal gradient of MS prevalence, with lower disease prevalence near the equator and an increasing prevalence in the Northern and Southern hemisphere, with 0.5–1.5 cases per 1000 inhabitants, compared to 0.2 cases per 1000 inhabitants in Latin America, Middle East and Africa [2,3]. MS typically appears in young adults and affects females more than twice as often as males [4]. The leading hypothesis is that MS is caused by a complex interaction between multiple genes and environmental factors, which leads to CNS inflammation, causing demyelination and axonal degeneration. To date, the best supported environmental risk factors in MS are a low serum level of vitamin D, Epstein-Barr virus (EBV) infection and smoking [5]. Genetic studies of MS all point to MS as a complex, polygenic disease, where several common genetic variants each exert a relatively small effect [6,7]. Gene identification is the first step in order to identify the biological pathways important for the disease. Characterization of these genes is crucial to facilitate the understanding of the molecular basis for MS pathogenesis.

## 2. Identification of MS Risk Loci

Already in 1972, the human leukocyte antigen (HLA) gene region was identified as a susceptibility locus for MS [8]. However, due to strong linkage disequilibrium (LD), it was difficult to clearly define the primary association. It is now well established that the HLA class II-DRB1 locus constitutes the primary genetic association, with the HLA-DRB1*15:01 allele as the major genetic risk factor for MS in most populations (odds ratio (OR) = 3.1) [9,10]. Furthermore, it has been convincingly shown that also HLA class I alleles have an effect independent of HLA-DRB1*15:01 [11,12], most strongly the HLA-A*02:01 allele, which is negatively associated with the risk for MS development (OR = 0.73) [10].

Phenotype-genotype studies of associated HLA alleles in MS have shown somewhat conflicting results, but a significant correlation was identified between age at onset of MS and the HLA-DRB1*15:01 allele [10]. Our recent analysis of the MS sub-phenotype, characterized by the presence of oligoclonal bands (OCB) in the cerebrospinal fluid, has shown association of HLA-DRB*04:04 with OCB negative MS and with HLA-DRB*15:01 more strongly in OCB-positive than OCB-negative MS [13].

In spite of intensive genetic research using both linkage studies and candidate gene approaches, it took close to 40 years before non-HLA loci were convincingly identified as associated with MS. In 2007, a moderately powered genome-wide association study (GWAS) identified the first non-HLA MS susceptibility loci, *i.e.*, the interleukin-7 receptor α (*IL7R*α) and *IL2R*α genes [14]. Simultaneously, the *IL7R*α gene was confirmed as an MS susceptibility gene in other cohorts [15,16]. In the following years, a series of GWAS- and meta-analyses were carried out, adding more regions to the list of confirmed MS associated loci, which in the spring of 2011 included 26 non-HLA genetic regions [6,17–26]. In August 2011, the largest GWAS in MS, including 9 772 MS cases and 17 376 controls, was published by the International Multiple Sclerosis Genetics Consortium (IMSGC) and the Wellcome Trust Case Control Consortium 2 (WTCCC2). This GWAS confirmed association to 23 of the previously suggested MS risk loci and identified an additional 29 novel susceptibility loci. Interestingly, the majority of these 52 MS-associated loci were located inside or close to genes of importance for the immune system, especially T-cell immunity [10]. These findings supported the notion that immunological mechanisms are crucial for MS development. When summarizing results from MS GWAS studies, as well as recent meta-analyses, close to 60 MS susceptibility genes have been identified by the end of 2012 [10,23,27,28]. Most of these variants are common and contribute only modestly to MS susceptibility (OR = 1.1–1.3) [7,10]. The list of MS susceptibility genes is expected to grow even further in the near future, when ongoing fine-mapping and replication studies will be completed.

A single nucleotide polymorphism (SNP) in the C-type lectin-like domain family 16A (*CLEC16A*) gene was among the genetic variants that showed suggestive association (rs6498169, *p* = 3.83 × 10^−6^) with MS in the first MS GWAS published in 2007 [14]. *CLEC16A* has thereafter been convincingly replicated as an MS susceptibility gene in a series of studies [29–37], including the large MS GWAS published in 2011 (rs7200786, *p* = 6.3 × 10^−14^, OR = 1.15) [10]. Of note, one-third of the identified MS loci have been reported to be associated with at least one additional autoimmune disease [7,10], and *CLEC16A* SNPs have shown association with several other autoimmune diseases (see Table 1). *CLEC16A* is preferentially expressed in immune cells [38], which also indicates that the CLEC16A molecule might play an important role in immune regulation. However, the function of CLEC16A is still not well defined. Interestingly, the *CLEC16A* gene is located at chromosome 16p13, in a region including several other immune-regulatory genes. Intensive efforts are ongoing, aiming to identify the implication of the identified *CLEC16A* associations and the functional impact on the encoded protein, as well as on its potential regulatory role on neighboring genes. In this paper, we review the current knowledge about this gene and the encoded molecule, which will serve as an example on how the field is moving from identification of MS susceptibility genes to functional molecular studies.

## 3. *CLEC16A*—An Autoimmune Candidate Gene

**Figure 1:**
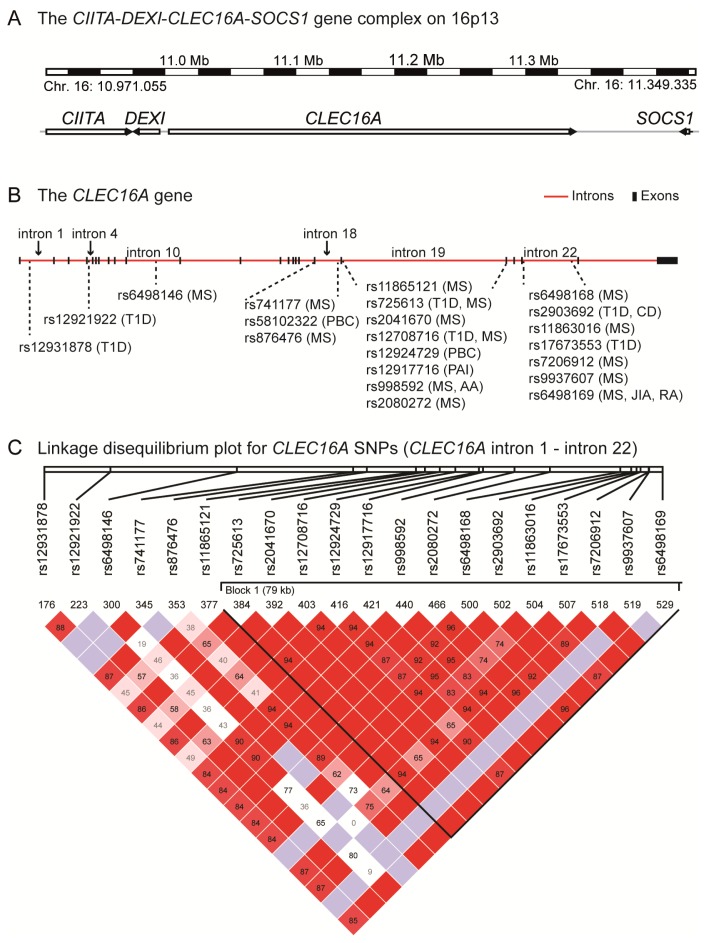
Schematic drawing of (**a**) the chromosome 16p13 genetic region comprising *CIITA*, *DEXI*, *CLEC16A* and *SOCS1* (Genome Reference Consortium Human Build 37 (GRCh37), chromosome 16: 10.971.055–11.349.335) and (**b**) the 238 kb *CLEC16A* gene (GRCh37, chromosome 16: 11.038.345–11.276.046), where the autoimmunity-associated single nucleotide polymorphisms (SNPs) and their localization are depicted; (**c**) Linkage disequilibrium plot (Haploview v. 4.2, CEU-population [46]) for the *CLEC16A* region (chromosome 16: 11.042.194 (*CLEC16A* intron 1)–11.249.329 (*CLEC16A* intron 22), GRCh37) harboring the autoimmune-associated SNPs indicated in (**b**).

The *CLEC16A* gene (previously known as KIAA0350; also designated Gop-1) is 238 kb, consists of 24 exons (http://www.ncbi.nlm.nih.gov/nuccore/NM_015226.2) [39] and is located on chromosome 16p13 (Figure 1A). In addition to MS, *CLEC16A* is associated with susceptibility to several autoimmune diseases (see Figure 1B and Table 1), including type 1 diabetes (T1D) [40], primary adrenal insufficiency (Addison’s disease) [41], Crohn’s disease [42], primary biliary cirrhosis [43], juvenile idiopathic arthritis [44], rheumatoid arthritis [35,44] and alopecia areata [45]. Many of the reported SNPs are in strong LD (Figure 1C). Therefore, it has been difficult to identify the primary genetic association in this gene region.

Common for the disease-associated *CLEC16A* SNPs is that they are located in intronic regions of *CLEC16A*, the majority in intron 19 and intron 22 of the gene (Figure 1B and Table 1). One rare variant, rs2241100 in exon 23 of the *CLEC16A* gene, results in a non-synonymous (ns) amino acid change at position 906 (G906E). To examine whether the T1D association could be due to the predisposing effect from this rare ns-*CLEC16A* SNP, Hakonarson and colleagues sequenced exon 23 in 20 T1D patients that were homozygous for the T1D rs2286973 risk SNP in *CLEC16A*. However, all individuals were homozygous for the common ns-rs224100 SNP [40]. Thus, no predisposition effect from the rare exon 23 variant was detected.

To validate *CLEC16A* as an MS susceptibility gene and to fine-map the association, 44 *CLEC16A* SNPs were genotyped in a collection of Australian samples. This study pointed towards rs6498169 in intron 22 as the most significantly associated *CLEC16A* SNP [34]. IMSGC reported that the *CLEC16A* rs12708716 SNP (in intron 19 of *CLEC16A* and in LD with rs6498169; D′ = 1, *R*^2^ = 0.259) was associated with MS at a genome-wide significance level [31]. We conducted a fine-mapping of the *CLEC16A* region in Norwegian samples, followed by replication of the top hits in two large Norwegian and British sample sets. Among the 57 SNPs tested, rs12708716 appeared to yield the superior association, followed by two other intronic *CLEC16A* SNPs; rs7206912 and rs6498169 [32]. Importantly, rs12708716 is in LD with the rs7200786 SNP (D′ = 1, *R*^2^ = 0.61) (Haploview v. 4.2, CEU-population [46]) in intron 19 of *CLEC16A* that later was confirmed to be associated with MS at a genome-wide significance level in the large GWAS published in 2011 [10].

## 4. The *CIITA*-*DEXI*-*CLEC16A*-*SOCS1* Gene Complex

The 16p13 chromosomal region where *CLEC16A* is situated has been the focus of several studies on MS, as well as other autoimmune diseases. Two immune-regulatory genes of potential interest for autoimmunity (see Tables 2 and 3), *i.e.*, the major histocompatibility complex (MHC) class II transactivator (*CIITA*) gene and the suppressor of cytokine signaling 1 (*SOCS1*) gene, are located close to *CLEC16A* (Figure 1A). *CIITA* encodes an essential transcription factor regulating gene expression of HLA class II molecules [57,58]. Due to the strong association of HLA-DRB*15:01 in MS, *CIITA* has been suggested as an MS candidate gene. Indeed, some reports have shown association between a *CIITA* promoter variant (rs3087456) and MS [59,60]. However, a multi-stage investigation did not confirm this association, but reported evidence for interaction between rs4774 (+1614 G/C missense mutation) and HLA-DRB1*15:01 in MS [61].

SOCS1 is a suppressor of cytokine signaling that is important for immune cell homeostasis and regulation of inflammation [67]. Gene variants in the 5′ untranslated region (UTR) of *SOCS1* (rs243324 and rs441349) have been identified in cytokine pathway gene screenings as MS susceptibility variants [66,68]. Furthermore, association to MS has been found for a SNP (rs243315) in the 5′ UTR of the protamine 1 (*PRM1*) gene [69], which is located downstream of this genomic region. However, this signal might be explained by strong LD between rs243315 and rs441349 within the 5′ UTR of the *SOCS1* gene (Haploview 4.2 [46]). Further evidence for involvement of *SOCS1* in MS comes from our recent study, showing that *SOCS1* expression is significantly lower in thymic tissue samples collected from children undergoing cardiac surgery, carrying at least one *CLEC16A* risk allele (at rs12708716, rs6498169 or rs7206912) compared to non-carriers of the risk allele [70]. Additionally, the dexamethasone-induced gene (*DEXI*), located between the *CLEC16A* and *CIITA* genes, has recently been suggested to be a novel autoimmune regulatory gene [70,71]. An expression quantitative trait locus for the *DEXI* gene was identified within intron 19 of *CLEC16A* in monocytes and in lymphoblastoid cell lines [71,72]. Additionally, *DEXI* expression was significantly lower in the above mentioned thymic tissue samples from individuals carrying the *CLEC16A* rs6498169 risk allele (in intron 22) compared to non-carriers of the risk allele [70]. The function of the protein encoded by *DEXI* is unknown; however, its transcript is induced by dexamethasone [73], which is a glucocorticoid analogue and an immunosuppressive drug. Of note, when *SOCS1* and *DEXI* expression was measured in whole blood samples from healthy controls genotyped for the same *CLEC16A* SNPs, we were not able to detect any correlation between gene expression and *CLEC16A* genotypes. This lack of correlation could be due to cell-specific, genetically determined variations that are overshadowed by the variation in the proportions of the various cell types within the blood samples. The discrepancies can also be caused by individual differences, as the whole-blood and thymic samples are harvested from different donor groups of different age and health states [70].

To clarify the role of the different MS-associated SNPs within the 16p13 chromosomal region harboring the *CIITA*-*DEXI*-*CLEC16A*-*SOCS1* gene complex, Zuvich and colleagues genotyped 149 SNPs in a combined American and British sample set and performed a detailed LD pattern and logistic regression analysis. Their data indicate that this region likely contains three independent MS disease loci; however, the *CLEC16A* rs7184083, which is in LD with rs12708716 (D′ = 1.00, *r*^2^ = 0.31), displayed the most significant *p*-value in this region [49]. Altogether, these data highlight the importance for further studies of this genetic region in relation to MS pathogenesis. Such efforts are already ongoing; for instance, in the “MS Immunochip project”, where IMSGC is genotyping samples from a large number of MS cases and controls using the “Immunochip”, a chip that was designed to replicate and fine-map disease risk loci associated with several autoimmune diseases [74–76]. Risk loci, among them the *CIITA*-*DEXI*-*CLEC16A*-*SOCS1* region (see Tables 1–3), are shared among autoimmune diseases [6]. With the established roles of *SOCS1* and *CIITA* in inflammation and autoimmunity [77,78] and the likely role of *CLEC16A* in immune regulation (see below), the refinement of the genetic association, as well as the molecular function, holds promises to elucidate the mechanisms behind autoimmune diseases.

## 5. *CLEC16A* Expression

Microarray expression data indicate that *CLEC16A* is extensively expressed in different immune cells, in certain parts of the brain, *i.e.*, cerebellum, spinal cord and pineal gland, and in testis [38,79]. Reverse-transcriptase polymerase chain reaction analyses have also detected low levels of *CLEC16A* expression in ovary and small intestine [80]. Laser scanning microscopy of the rat brain showed CLEC16A protein expression in astrocytes and neurons, but not in microglia. *CLEC16A* expression was further increased in rat astrocytes upon intraspinal injection of lipopolysaccharide (LPS) [81].

The human *CLEC16A* gene has been suggested to encode at least three different splice variants [79], two long isoforms expressed from all 24 exons or from 21 exons and a short transcript encoded from the last four exons. The two long *CLEC16A* isoforms are expressed in whole blood and in thymic tissue samples [32]. Unpublished data from our laboratory further show expression of these two long isoforms in a wide range of primary immune cells, as well as in immune cell lines and in the HEK293T cell line (a human embryonic kidney cell line). The third isoform was not tested in these analyses. Normal genetic variation may contribute to disease susceptibility and severity by affecting splicing of mRNA [82]. Interestingly, the relative expression of the two long *CLEC16A* isoforms did correlate with the MS associated, intronic rs12708716 variant in human thymic tissue samples. However, when whole blood samples, which are heterogeneous in their cellular composition, were analyzed, no such correlation was found [32]. This indicates that the relative expression of *CLEC16A* isoforms could be cell type specific.

**Figure 2:**
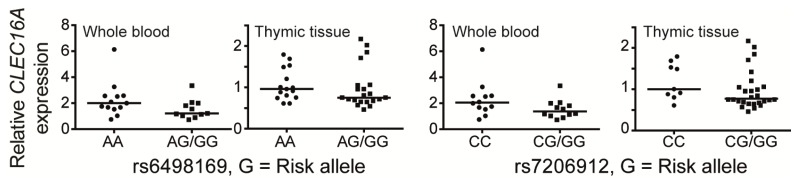
The graphs show relative expression of total *CLEC16A* (including the two long isoforms) normalized to the TATA-binding protein (*TBP*) gene in thymic tissue samples from 37 Norwegian children under the age of 13 undergoing corrective cardiac surgery and whole blood samples from 24 healthy, normal controls genotyped for the indicated SNPs in *CLEC16A* intron 22, as described [70]. Individuals carrying the risk allele were compared with individuals homozygous for the protective allele. Left graphs: rs6498169 (risk allele G): GG/AG: *n* = 21 (thymus), *n* = 11 (whole blood), AA: *n* = 15 (thymus), *n* = 13 (whole blood); right graphs: rs7206912 (risk allele G): GG/CG: *n* = 28 (thymus), *n* = 12 (whole blood), CC: *n* = 9 (thymus), *n* = 12 (whole blood). Correlation between gene expression levels and genotypes were assessed by Mann-Whitney U-test (GraphPad Prism 5, GraphPad Software, Inc., San Diego, CA, USA) and were found to be non-significant. Horizontal lines indicate the median values within the groups.

The fact that non-coding, disease associated *CLEC16A* SNPs might affect *CLEC16A* expression in a cell-type specific manner has also been suggested for the non-coding rs2903692 (in strong LD (*r*^2^ = 0.88) with rs12708716), which shows genome-wide significant association with T1D. Investigation of a synonymous exon 19 SNP (rs2286973, a marker for the intronic T1D rs2903692 risk allele) did not reveal genotype dependent differences in *CLEC16A* expression in ten lymphoblastoid cell lines. However, when *CLEC16A* expression was analyzed in four different natural killer cell lines, a trend towards higher *CLEC16A* expression was observed in the cell line that was homozygous for the A-allele in rs2903692 [40]. When we analyzed the expression of total *CLEC16A* (including the two long isoforms) in relation with *CLEC16A* genotype (rs12708716, rs6498169 and rs7206912), we did not find any significant correlation, neither in whole blood, nor in thymic tissue samples [32,70]. However, we did observe a trend towards lower *CLEC16A* expression in the presence of an MS associated risk SNP at either rs6498169 or rs7206912 (Figure 2) [70]. Lack of power could explain the absence of a significant correlation; therefore, *CLEC16A* expression analyses in increased numbers of samples are needed.

Of note, when sorting the samples according to the rs12708716 genotype (in *CLEC16A* intron 19), we did not reveal any sign of genotype-dependent *CLEC16A* expression in thymic samples [70]. However, no such comparison could be analyzed in whole blood, as only one whole blood sample was homozygous for the protective rs12708716 G-allele. In conclusion, *CLEC16A* expression analysis in purified immune-cell subsets will be important to evaluate a potential cell specific regulation of *CLEC16A*.

## 6. The Structural Domains of the CLEC16A Molecule

**Figure 3:**
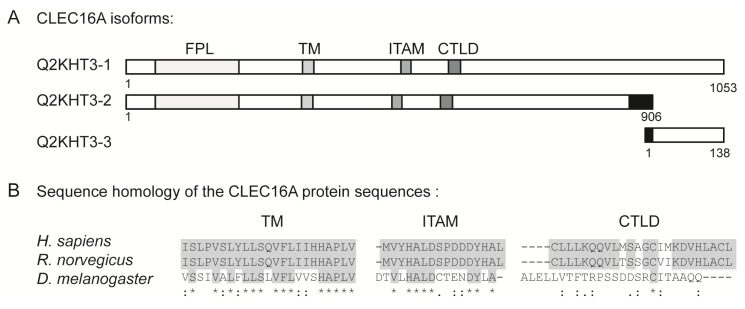
(**a**) Schematic representation of proposed CLEC16A protein variants (Q2KHT3-1, -2 and -3) highlighting from the *N*-terminal: a highly conserved uncharacterized protein domain, the FPL motif (amino acid (aa) 51–199 in Q2KHT3-1 and Q2KHT3-2,), a predicted transmembrane (TM) domain (aa 308–330 in Q2KHT3-1), an immunoreceptor-tyrosine based activation motif (ITAM, aa 483–499 in Q2KHT3-1) and the C-type lectin-like domain (CTLD, aa 566–588 in Q2KHT3-1). Black boxes indicate sequences where the amino acid identity differs from the canonical sequence; (**b**) Protein sequence alignment of the transmembrane region (TM, left), the immunoreceptor tyrosine-based activation motif (ITAM) sequence (middle) and the CTLD (right) of CLEC16A from *Homo sapiens*, *Rattus norwegicus* and *Drosophila melanogaster*. Boxed grey areas highlight fully conserved amino acids between the indicated species. “*” indicates identical amino acids in all three sequences; “:” indicates conserved substitution of amino acids; “.” indicates semi-conserved substitution of amino acids; and open space indicates no conservation of amino acid between the three species.

The full-length *CLEC16A* gene encodes a protein of 1053 amino acids (Q2KHT3-1, see Figure 3A), whose name was designated C-type lectin-like domain family 16A (CLEC16A) due to the presence of a C-type lectin-like domain (CTLD). Interestingly, other members of the C-type lectin family, such as DCIR (for dendritic cell immunoreceptor [83]) have been linked to autoimmunity in rodent models [84]. The most common CTLD function in vertebrates, and the ancestral mode of action, is Ca^2+^-dependent carbohydrate binding. Consequently, these proteins are involved in diverse processes, such as cell-cell adhesion, plasma glycoprotein turnover, as well as innate pathogen recognition [85]. The short nature of the CLEC16A CTLD (only 23 amino acids [86]), makes it unlikely that the CLEC16A protein region harboring this CTLD can fold properly into the compact 110–130 amino-acid domain of a carbohydrate recognition element [85,87,88]. However, many CTLDs have evolved to specifically recognize other ligands, such as proteins, lipids, as well as inorganic ligands [87]. Whether CLEC16A can interact with other potential ligands through its CTLD has not yet been studied.

Upon ligand binding, C-type lectin receptors within the innate immunity commonly induce multiple signal transduction cascades through their own immunoreceptor tyrosine-based activation motifs (ITAMs) or through ITAMs of interacting proteins [89]. Interestingly, CLEC16A contains a potential ITAM ([90,91]; amino acids (aa) 483–499, see Figure 3A), thereby indicating a role for CLEC16A in immune-cell signaling. In addition to the CTLD and the ITAM, CLEC16A contains an *N*-terminally, highly conserved and uncharacterized domain, designated FPL (pfam09758) [92] (aa 51–199) and it is predicted to contain a trans-membrane region [93] (aa 308–330). As can be seen from the schematic drawing of CLEC16A in Figure 3A, these four domains are intact in the truncated CLEC16A isoform of 906 amino acids (Q2KHT3-2). The sequence of this isoform differs from the canonical sequence in that the following regions are missing; aa 201–202 (encoded by exon 6), aa 419–434 (encoded by parts of exon 11–12), as well as aa 925–1053 (encoded by the last two exons). Additionally, amino acids corresponding to aa 882–924 (exon 23) in Q2KHT3-1 differ in identity between the two isoforms [94]. Furthermore, a third transcript encoding a protein of only 138 amino acids, designated Q2KHT3-3, is listed at http://genome.ucsc.edu [79]. This splice variant is encoded from the four last exons of the *CLE16A* gene and lacks amino acids 1–913. In addition, the amino acids 914–935 differ from the canonical sequence [94,95], and this short isoform lacks the motifs that are identified in Q2KHT3-1 and -2.

## 7. The Function of CLEC16A

Up to this date, there is to our knowledge no published work on the functional aspects of human CLEC16A. However, two recent papers report studies of the CLEC16A orthologue, Ema, in *Drosophila melanogaster*[96,97]. Kim and colleagues have demonstrated that Ema is an endosomal protein with an important role in endosomal maturation and trafficking. They demonstrated that absence of Ema in the *Drosophila* garland cells induced accumulation of large endosomal intermediates, disrupted membrane trafficking and failure of proper lysosomal degradation [96]. In addition, Ema has been suggested to promote membrane traffic from the Golgi apparatus to the autophagosomes, since it is required for autophagosomal growth and efficient autophagy [97]. As there is 41% amino acid identity between human CLEC16A and Ema (clustalW [98]), these studies could pinpoint relevant functions also for the human counterpart. Interestingly, human CLEC16A can rescue the membrane-trafficking defects in Ema-mutant fly [96], and it restores normal autophagosomal growth when expressed in Ema-mutant fat body cells [97]. This suggests that Ema and CLEC16A are functionally conserved. The CLEC16A CTLD is not conserved from human to *Drosophila*, and Ema does not contain an ITAM consensus sequence (YXXI/LX6–8YXXI/L) (see Figure 3B) [90,91]. Of note, the FPL motif is well-conserved between human and *Drosophila* CLEC16A (clustalW [98]) and the putative transmembrane domain of Ema (Tmpred at http://www.ch.embnet.org) [93] lies in a highly conserved region of the protein (Figure 3B and data not shown), indicating that these two domains may be of importance for the conserved functions reported by Kim and colleagues [96,97].

The endolysosomal pathway regulates the levels and distribution of membrane receptors [99]. The study of Kim and colleagues [96] suggests that CLEC16A might confer susceptibility to autoimmune disorders through its role in endosomal regulation of immune receptor signaling pathways, leading to aberrant immune responses of autoreactive T-cells. Furthermore, its endosomal localization within the cell suggests that it might affect antigen presentation by the HLA-complex, which could affect T-cell immune responses in the periphery by autoreactive T-cells, as well as establishing self-tolerance during thymic T-cell development. Interestingly, CLEC16A shares localization to endosomal structures with molecules encoded by other MS susceptibility genes, such as signal transducer and activator of transcription 3 (STAT3) [100], tyrosine kinase 2 (Tyk2) [101] and ectopic viral integration site 5 (Evi5) [102,103].

Autophagy is a lysosomal catalytic process for bulk degradation of unwanted cytoplasmic substances and for recycling of nutrients. Autophagy is also involved in innate and adaptive immune responses, playing an important role for combating microbes, in antigen processing for MHC presentation and in lymphocyte development, survival and proliferation. Furthermore, interfering autophagy-related processes have been implicated in several diseases, including autoimmunity [104]. A number of GWAS analyses have linked SNPs in autophagy-related genes to susceptibility for autoimmune diseases, e.g., autophagy-specific gene 1 (*ATG5*) in systemic lupus erythematosus [105] and unc-51-like kinase 1 (*ULK1*) in Crohn’s disease [106]. A potential role for *CLEC16A* in autophagy would add this gene to the list of autophagy-related autoimmune associated genes. Furthermore, it has been reported that a couple of MS risk loci might play a role in autophagy; *CYP27B1* expression is required for TLR2/1 mediated antibacterial autophagy in monocytes [107], whereas CD40 triggers killing of *Toxoplasma gondii* in an autophagy-dependent manner [108].

Inflammation leading to demyelination and axonal damage in the CNS is the main pathological feature of MS. During CNS inflammation, glial cells are activated and secrete cytokines. Astrocytes are the major constituents of the glial cell population in the CNS and do not only have the ability to enhance immune responses and inhibit myelin repair, but they can also limit CNS inflammation by supporting oligodendrocyte and axonal regeneration [109]. Interestingly, Wu and colleagues were able to show that CLEC16A is important for astrocyte activation in rats, as LPS-induced production of the pro-inflammatory cytokine, tumor necrosis factor alpha (TNFα), was reduced in astrocytes when *CLEC16A* expression was knocked down after transfection with CLEC16A-specific small-interfering RNA [81]. Taken together, this suggests that CLEC16A might be of importance during CNS inflammation, at least in a rat model system.

## 8. The *CLEC16* Gene Region and Disease Susceptibility

In summary, fine-mapping analyses of MS associated SNPs in the 16p13 region point to *CLEC16A* SNPs as the most strongly associated MS genetic variants in this gene-rich region [49]. However, as these SNPs are intronic, we do not know whether CLEC16A *per se* is important for development of MS or whether these SNPs are expression quantitative trait loci for *CLEC16A* or other genes, if they tag a causal rare SNP or if they are in LD with other regulatory SNPs in the same genetic region. Non-coding SNPs may confer susceptibility to disease development by affecting expression of nearby genes in *cis*[110,111]. Indeed, MS associated SNPs in the *CLEC16A* region have been shown to correlate with expression of the neighboring *SOCS1* and *DEXI* genes [70,71]. Interestingly, chromosome conformation capture assays performed by Davison *et al.*[71] proposed that a loop can be formed between the *DEXI* promoter and a 20 kb region of *CLEC16A* intron 19 (containing the MS associated rs12708716) shown in three tested cell lines (THP-1 (a monocytic cell line), A549 (a lung epithelial cell line) and a human EBV-transformed B-cell line). This supports that intronic, disease-associated SNPs in *CLEC16A* may be in physical proximity with a distant gene, such as *DEXI*, thereby potentially affecting the binding of transcription factors or other regulatory proteins important for gene expression. The same close proximity was not observed between the *SOCS1* gene and the intron 19 of *CLEC16A* in these cell lines [71].

*CLEC16A*, *SOCS1* and *DEXI* are co-expressed in thymic tissue samples [70] and in human lymphoblastoid cell lines [49], but not in whole blood [70] nor in monocytes [71]. This cell-specific co-regulation could be due to a common, regulatory element controlling cell-specific expression of all three genes. In support of this notion, the co-expressed *DEXI*, *CLEC16A* and *SOCS1* genes constitute an “active” chromatin domain, at least in lymphoblastoid cells, as analyzed by methylation of lysine 27 on histone H3 and CCCTC-binding factor (CTCF) occupancy [49,79]. If one or more autoimmune-associated SNPs are in LD with a SNP in a common regulatory region, it could affect gene expression of several genes in this region, including *DEXI*, *CLEC16A* and *SOCS1*, meaning that all three genes might have an impact on disease susceptibility or phenotype.

Gene identification is an important first step to pinpoint a relevant gene region; however, GWASs do not tell whether the disease-associated variant is causal. By combining GWAS data with data from the Encyclopedia DNA Elements (ENCODE) project [112] for a number of traits and diseases, Schaub and colleagues were able to show that, for the majority of associations, the SNP most likely to play a functional role is usually a different SNP in strong LD with the reported association [113]. In fact, in MS, it was recently published that a majority of MS-associated SNPs identified in GWASs were located in functionally active DNA sites, so-called DNase hypersensitive sites. Interestingly, this co-localization was more pronounced in immune cells that are relevant for MS pathogenesis compared to other cell types [114]. After finalizing the fine-mapping of this genetic region, it will be possible to combine these results with the ENCODE data to define functional DNA elements that will be interesting for functional studies of this gene and neighboring genes in the time to come.

## 9. Conclusions

*CLEC16A* has been convincingly replicated as an MS susceptibility gene in a series of studies (see Table 1). In the gene-rich region of chromosome 16p13, several autoimmune disease-associated SNPs have also been identified in the neighboring *SOCS1* and *CIITA* genes, as well as in intergenic regions (see Tables 2 and 3). However, SNPs within intronic regions of *CLEC16A* appear to display the strongest association, at least in MS [49]. Together with its selective expression in immune cells [38] and due to its association with a wide variety of autoimmune diseases, the *CLEC16A* gene and the encoded molecule is indeed of interest for further functional studies. In addition, as intronic *CLEC16A* sequences, harboring disease-associated SNPs, are correlated with expression of neighboring genes, *i.e.*, *SOCS1* and *DEXI*[70,71], the interaction between the *CLEC16A* gene and these genes needs to be further analyzed to understand new pathological mechanisms for development of autoimmunity.

## Figures and Tables

**Table 1:** *CLEC16A* SNPs associated with autoimmune diseases. The table displays published *CLEC16A* SNPs that have shown association with indicated autoimmune diseases in different cohorts. Crohn’s = Crohn’s disease; Addisons = Addisons disease; A. Areata = Alopecia Areata; Aus = Australia; Bel = Belgium; Can = Canada; Eur = Europe; Fin = Finland; Nor = Norway; Rom = Romania; Swe = Sweden.

Disease	SNP	Intron	Cases/Controls/Trios	Subject cohort	Reference
Multiple Sclerosis	rs6498168	22	2322/5418/1540	UK, USA	[14]
	rs6498169	22	1146/1309	Australia	[30]
	rs725613	19	1498/1706	Sardinia	[33]
	rs12708716	19	5737/10296/2369	Aus., Bel., Nor., Swe., UK, USA	[31]
	rs6498169	19			
	rs6498146	10			
	rs741177	18	1146/1309	Europe	[34]
	rs876476	18			
	rs11863016	22			
	rs9937607	22			
	rs11865121	19	2624/7220	UK, USA	[23]
	rs6498169	22	435/550	Spain	[35]
	rs6498169	22	211/182 (+521 multiplex controls)	UK	[36]
	rs6498169	22	1853/2128	Holland, Can.	[29]
	rs12708716	19	918/656	USA (afro American)	[37]
	rs6498169	22			
	rs2080272	19			
	rs2041670	19	603/825	Europe	[47]
	rs998592	19			
	rs12708716	19	197/197	India	[48]
	rs12708716	19			
	rs7206912	22	3102/5047/1113	Nor., UK	[32]
	rs6498169	22			
	rs7184083	22	1343/1379	UK, US	[49]

Type 1 Diabetes	rs12708716	19	2000/3000	UK	[50]
	rs725613	19			
	rs2903692	22	1896/1146/873	Eur., USA, Aus., Can.	[40]
	rs17673553	22			
	rs12708716	19	4000/5000/2997	UK, USA, Fin., Nor., Rom.	[26]
	rs725613	19	1037/1706	Italy (Sardinia)	[33]
	rs2903692	22	735/621	Japan	[51]
	rs725613	19	205/422	China (Han)	[52]
	rs6498169	22	316/550	Spain	[35]
	rs12921922	4	131/121	China	[53]
	rs12931878	1			
	rs12708716	19	1212/2513	Germany	[54]
	rs2903692	22	1743/790	Japan	[55]

Rheumatoid arthritis	rs6498169	22	600/550	Spain	[35]
	rs6498169	22	1318/2149	Norway	[44]
	rs6498169	22	600/550	Spain	[35]

Primary biliary cirrhosis	rs58102322	18	1450/2967	Europe	[43]
	rs12924129	19			
	rs12924729	19	2460/7677	UK	[56]

Crohn’s	rs2903692	22	1264/890	Spain	[42]

Addisons	rs12917716	19	542/1220	Norway, UK	[41]

A. Areata	rs998592	19	1702/1723	Europe	[45]

**Table 2:** *CIITA* SNPs associated with autoimmune diseases. The table displays published *CIITA* SNPs that have shown association with indicated autoimmune diseases in different cohorts.

Disease	SNP	SNP localization	Cases/Controls/Trios	Subject cohort	References
Multiple sclerosis	rs4774 1	Exon 11	1320/1363	Europe	[61]
	rs4774	Exon 11	1343/1379	UK, US	[49]
	rs3087456	Promoter	548/528	Scandinavia	[59,60]

Type 1 Diabetes	rs11074932	Promoter	5 cohorts	Sweden	[62]
	rs3087456	Promoter			

Rheumatoid arthritis	rs3087456	Promoter	1288/709	Scandinavia	[60]
	rs3087456/ rs4774 2	Promoter/ exon 11	580/454	Spain	[35]
	rs8048002	Intron 3	3551/4827	Norway, Sweden	[63]
	rs3087456	Promoter			

SLE	rs4774	Exon 11	1342/4301	Europe	[64]

Addisons disease	rs8048002	Intron 3	542/1220	Norway, UK	[41]
	rs3087465	Promoter	128/406	Italy	[65]

1 Significant in the presence of HLA-DRB1*15:01.

2 rs3087456G/rs4774C haplotype is associated with indicated disease.

SLE = systemic lupus erythematosus.

**Table 3:** *SOCS1* SNPs associated with autoimmune diseases. The table displays published *SOCS1* SNPs that have shown association with indicated autoimmune diseases in different cohorts.

Disease	SNP	SNP location	Cases/Controls	Subject cohort	Reference
Multiple sclerosis	rs1640923	Downstream	1343/1379	UK, US	[49]
	rs12922733	Upstream			
	rs243324	Promoter	3919/4003	Europe, USA	[66]

Primary biliary cirrhosis	rs243325	Promoter	1450/2967	Europe	[43]
